# Association of Shadowing Program for Undergraduate Premedical Students with Improvements in Understanding Medical Education and Training

**DOI:** 10.7759/cureus.6396

**Published:** 2019-12-16

**Authors:** Christine Thang, Natalie M Barnette, Kunal S Patel, Courtney Duong, Dillon Dejam, Isaac Yang, James H Lee

**Affiliations:** 1 Pediatrics, University of California, Los Angeles, Los Angeles , USA; 2 Pediatrics, University of California, Los Angeles, Los Angeles, USA; 3 Neurosurgery, University of California, Los Angeles, Los Angeles, USA; 4 Neurosurgery, David Geffen School of Medicine at University of California, Los Angeles, Los Angeles, USA; 5 Neurosurgery, David Geffen School of Medicine, University of California, Los Angeles, Los Angeles, USA

**Keywords:** undergraduate medical education, shadowing

## Abstract

Objective: Physician shadowing has become ubiquitous to the premedical experience. However, students without connections to a medical professional are oftentimes forced to reach out to physicians independently from a program. Subsequently, these inquiries may go unanswered as they oftentimes appear unsolicited. The primary goals in the design and development of our program were to increase access to a clinical observership experience at our academic institution utilizing resident physicians as primary supervisors.

Methods: In January 2017, the Educational Shadowing Program (ESP) was established at our institution wherein undergraduate students could shadow within the Pediatric Continuity Clinic (PCC) staffed by pediatric resident physicians. ESP undergraduates shadowed the residents as they performed their history taking and physical exams and as they presented their patients to the attending physicians. Between patient encounters, the students assisted the residents in their administrative work which was completed as needed. ESP students were surveyed at their first orientation meeting and during the final case conference.

Results: The pre-participation survey showed that none of the student participants strongly agreed to having a good understanding of what the job of a resident physician entails. By the end of their 30 weeks, the proportion of participants with a strongly perceived understanding increased significantly. The proportion of student respondents that strongly agreed with their understanding of the physician-patient interaction also improved significantly over the study period, from 33% to 78%. Seventy-two percent of the residents surveyed agreed or strongly agreed that they enjoyed having the undergraduates in the clinic, affirming the positive effects of the program on the resident physicians. Forty-five percent of residents agreed or strongly agreed that the undergraduates improved their workflow in the clinic.

Conclusion: This study demonstrates that establishing an undergraduate shadowing program in a busy academic pediatric clinic that involves resident physicians can be an overall positive experience for all participants. Fostering premedical student interest in pediatric care and primary care can possibly lead to more physician commitment to these fields, potentially helping to alleviate impending physicians in these specialties.

## Introduction

Physician shadowing has become ubiquitous to the premedical experience. Medical schools frequently look for shadowing experiences among applicants as a surrogate marker for the students’ enthusiasm for medicine and their practical awareness of what being a physician entails [1-3]. Premedical students are expected to demonstrate some experiential learning in the clinical setting, and the means by which they meet this expectation is typically by shadowing physicians. Thus, programs offering such shadowing opportunities are highly coveted by premedical students [2,4].

The benefits of shadowing for premedical students are two-fold: students are able to observe the realities of their potential career while simultaneously acquiring insight into the medical field [1]. The opportunity to observe patient-physician interactions, procedures, and examinations thus becomes highly sought after by premedical students [5]. Premedical students who have shadowed a physician are more confident about their goals and secure in their career choice [1-3,6]. Further along, those who have shadowed prior to entering medical school perceive themselves in better anticipation of the academic rigor demanded by the medical school curriculum [2,4,7-8]. 

In addition to benefiting premedical students and impacting their career path, shadowing has been demonstrated to have a positive impact on the patient experience as well. Bing-You et al. reported that 21.9% of patients in a family practice setting agreed that student observers had a positive effect on their visit, while the other 78.1% considered it a neutral effect on their visit [3]. In the same study, 96.9% of patients stated that they would welcome a student to shadow the physician during their next visit. Authors speculate from patient interviews that patients feel a sense of fulfillment from the idea that they are contributing to the education of future physicians [3,9-11].

The shortcomings of shadowing have also been detailed, particularly with respect to problems in access and structure. Premedical students with connections to the medical community are more easily able to secure physician-mentored experiences. Those students without connections are oftentimes forced to reach out to physicians independently from a program. These communications like emails appear unsolicited to the targeted mentor and may go unacknowledged. With respect to structure, the “educational direction is unspecific: students are simply advised to arrive at their shadowing site and take notes [12].” The literature describes multiple examples of undergraduate shadowing experiences for students pursuing medicine and for medical students developing their clinical skills [13]. Few studies have investigated a formal longitudinal shadowing program structured around undergraduate students and resident physicians. The potential benefits of developing a shadowing program with undergraduate students and resident physicians, rather than faculty, include: less formality leading to more open conversations, a smaller age divide allowing for more shared and similar experiences, and resident physicians gaining teaching experience and beginning to integrate teaching into their practice.

In recognizing the benefits and challenges of a clinical shadowing program, the primary goals in the design and development of our program were to increase access to a clinical observership experience at our academic institution and to create a sustainable program benefiting both undergraduate shadowers as well as resident physicians. After the inaugural year, the program was evaluated by qualitative and quantitative surveys assessing the perceptions of the premedical students’ shadowing experiences and the reciprocal experience for the physician trainees. In this study, we describe the structure of our resident-student shadowing program and evaluate its effect on both parties.

## Materials and methods

In January 2017, the Educational Shadowing Program (ESP) was established at the University of California, Los Angeles (UCLA) wherein undergraduate students could shadow within the Pediatric Continuity Clinic (PCC) staffed by pediatric resident physicians and their attending physicians. The academic-based outpatient clinic provides both urgent and well child care for general pediatrics and medical home pediatric patients. The clinic is staffed by trainees of the UCLA pediatric residency program comprised of 101 residents who rotate through the clinic in two-week service intervals and during weekly half-day continuity clinics. To meet institutional regulations, the ESP was registered with the UCLA Volunteer Services Department, and the study was approved by the university’s Institutional Review Board. Undergraduate students from the UCLA American Medical Student Association (AMSA) Pre-Medical Chapter were recruited to volunteer for the program. In a formal selection process conducted by undergraduate student leadership, student observers were selected based on their written application that detailed their level of shadowing interest and commitment to the program. Acceptance to the program was not contingent on participation in this research project. All undergraduates completed required health screenings and hospital compliance trainings. Undergraduates also received volunteer hours for their participation. Each student was scheduled for a weekly four-hour shadowing shift over the 30 weeks of the academic year.

Pediatric residents were approached at the beginning of their clinical rotation to determine their willingness to participate as an ESP shadowing mentor. The shadowing and administrative roles of the ESP participants were explained at this time. One first-year and one second-year pediatric resident rotates through PCC every two weeks. ESP participants would introduce themselves to the pediatric trainees at the start of every shift and shadow them during that clinical half-day. Residents were encouraged to involve the observers in both clinical and administrative activities of that clinic day. ESP undergraduates shadowed the residents as they performed their history taking and physical exams and as they presented their patients to the attending physicians. Between patient encounters, the students assisted the residents in their administrative work including completing patient-care related forms, faxing documents, scanning outside medical records, printing patient educational handouts, and completing other non-physician level tasks as needed. At the end of each 10-week academic quarter, all ESP observers participated in a mandatory educational case conference. Each undergraduate was assigned to present a patient case from their PCC shadowing experience. A standardized template was provided to direct their preparation for the presentation. A resident and attending physician at the case conference would then integrate teaching points from each case presentation into the educational conference.

The inaugural year of ESP and its first cohort of undergraduate shadowers began in October 2017 and finished in June 2018. ESP students were surveyed at their first orientation meeting and during the final case conference. Every participant completed a one-page printed questionnaire. A pre-participation seven-item questionnaire collected demographic data, quantified medical career interest, and queried baseline understanding of resident responsibilities and pediatric clinic activities. Subsequently, a ten-item post-participation questionnaire (seven identical statements from the pre-participation survey and three additional program evaluation questions) was completed by the undergraduates at the end of the 30-week program. The pediatric residents also anonymously and voluntarily submitted a single page five-item questionnaire at the end of their two-week PCC clinical rotation. For each of the questionnaires, respondents rated their level of agreement with each statement on a five-point Likert scale. Qualitative information from open-ended questions was also collected.

Student survey data was analyzed using paired t-test comparing changes for each statement’s mean score pre- and post-shadowing. Statistical significance was set at a *p* < 0.05 for each analysis.

## Results

In its first year, fifteen UCLA undergraduates applied to ESP, and ten students were selected to participate in the program. Table 1 summarizes the demographic data for the pilot undergraduate student cohort. Eight students identified as female, and two students identified as male. The undergraduate cohort consisted of seven fourth-year, one third-year, and two second-year college students. The mean age was 20.1 years, ranging from 19 to 21 years old. Among the ten undergraduate student participants, one female student was unavailable to complete the post-participation survey and thus was excluded in the pre- and post-participation survey data analysis. Eleven UCLA pediatric resident trainees agreed to and subsequently completed the program evaluation questionnaire.

**Table 1:** Demographic data for undergraduate participants (N=10)

Response	n (%)
Gender	
Male	2 (20)
Female	8 (80)
Undergraduate Year	
Second	2 (20)
Third	1 (10)
Fourth	7 (70)
Major	
Psychobiology	7 (70)
Psychology	1 (10)
Biology	1 (10)
Neuroscience	1 (10)
Prior shadowing experience	
Formal program	1 (10)
Informal with a physician	2 (20)
Informal (i.e. scribe, medical volunteer trips)	6 (60)
None	1 (10)

Table 2 summarizes pre- and post-questionnaire ratings of the undergraduate participants. Prior to the shadowing experience, few students responded positively (agreed or strongly agreed) to questions elucidating their understanding of pediatric-specific clinical activities including the details of a well child check (33%) and the workflow of a pediatric clinic (33%). Comparatively, at program entry, 67% of students agreed that they were familiar with what happens in an urgent care visit. From comparing the pre- and post-participation survey means for the three statements addressing familiarity with the pediatric clinic flow, a well child check, and an urgent care visit, all demonstrated statistically significantl increase in statement agreement (Table 2).

**Table 2:** Student survey responses (N=9) SD = strongly disagree, D = disagree, Neu = neutral, A = agree, SA = strongly agree; Pre = pre-shadowing survey responses, Post = post-shadowing survey responses; *= statistical significance of  *p* < 0.05 from paired t-test comparison of the mean pre- and post- surveys. N = 9 since one of the ten undergraduate program participants was excluded due to the inability to complete post-survey.

Response	Pre or Post	1, 2 SD,D %	3 Neu %	4 A %	5 SA %
Clinical/Provider Activities					
I understand how physicians interact with patients in a clinical setting* (p=0.03)	Pre			67%	33%
	Post			22%	78%
I am familiar with what happens in an urgent care visit* (p=0.04)	Pre		22%	46%	22%
	Post			33%	67%
I am familiar with what happens in a well child check* (p=0.009)	Pre	45%	22%	33%	
	Post		11%	22%	67%
I have a good understanding of what the job of a resident physician entails* (p=0.04)	Pre		22%	78%	
	Post			56%	44%
I understand how a pediatric clinic functions* (p=0.005)	Pre	11%	56%	33%	
	Post			56%	44%
Career Questions					
I have a good understanding of what it takes to be a successful candidate in applying to medical school	Pre		22%	56%	22%
	Post		11%	56%	33%
I am interested in pursuing a career in medicine	Pre			11%	89%
	Post				100%
Program evaluation					
This program has been effective in increasing my exposure to residents	Post				100%
This program has been effective in increasing my clinical exposure to pediatrics	Post				100%
As a result of this program, I am more interested in pursuing a career in medicine	Post			11%	89%

Two survey questions were directed at the participants’ understanding of the provider’s activities; one for the resident trainee role and the second addressed the observed physician-patient interactions (Table 2). Results for the pre-participation survey showed that none of the student participants strongly agreed to having a good understanding of what the job of a resident physician entails. By the end of their 30 weeks of shadowing, the proportion of participants with a strongly perceived understanding increased significantly. The proportion of student respondents that strongly agreed with their understanding of the physician-patient interaction also improved significantly over the study period, from 33% to 78%. Notably, for the entire cohort, the mean scores (pre- and post-) for this survey item increased, indicating improved understanding of how physicians interact with patients. 

No statistically significant differences in agreement were seen in other pre- and post-statements for non-clinical questions. The survey item targeting the understanding of what it takes to be a successful candidate applying to medical school had a baseline agreement of 78% and post-survey value of 89%. The number of respondents with a strong interest in pursuing a career in medicine was high on the pre-survey at 89% as well as on the post-survey at 100%.

Within the undergraduate student post-participation survey, three additional five-item Likert scale questions were included to evaluate aspects surrounding the program’s perceived general successes (Table 2). All students strongly agreed in the effectiveness of the ESP in increasing their clinical exposure not only to pediatrics, but also to resident physicians. Nearly all students strongly agreed (89%) that they had become more interested in pursuing a career in medicine as a result of this program.

From the resident physicians’ survey responses (Table 3), there was both quantitative and qualitative feedback supporting the undergraduate ESP. Seventy-two percent of the residents surveyed agreed or strongly agreed that they enjoyed having the undergraduates in clinic with them, affirming the positive effects of the program on the resident physicians. In terms of teaching and mentoring the ESP participants, 45% of resident physicians agreed or strongly agreed that they were able to teach the undergraduates during their time together, but only 36% of resident physicians agreed or strongly agreed that they were able to mentor the undergraduates during their time together. Forty-five percent of residents agreed or strongly agreed that the undergraduates improved their workflow in clinic. However, only 36% of resident physicians agreed or strongly agreed that they were able to discuss a career in medicine with the ESP participants.

**Table 3:** Pediatric resident survey responses (N=11) SD=strongly disagree, D=disagree, Neu=neutral, A=agree, SA=strongly agree

Response	1, 2 SD,D n (%)	3 Neu n (%)	4 A n (%)	5 SA n (%)
I enjoyed having undergraduate students in the clinic with me.	1 (9)	2 (18)	3 (27)	5 (45)
I was able to teach the undergraduate students in the clinic.	2 (18)	4 (36)	3 (27)	2 (18)
I was able to mentor the undergraduate students in the clinic.	3 (27)	4 (36)	3 (27)	1 (9)
The undergraduate students helped me complete my workflow in the clinic.	3 (27)	3 (27)	3 (27)	2 (18)
I was able to discuss a career in medicine with undergraduate students.	5 (45)	2 (18)	2 (18)	2 (18)

## Discussion

The results of this study demonstrate that establishing an undergraduate shadowing program in a busy academic pediatric clinic that involves resident physicians can be an overall positive experience for all participants. It has been recognized that near-peers serve as effective mentors with better social and cognitive congruence between the mentee and the mentor [1-2,4,7-8]. While residents seek faculty support for their clinical and scholarly activities, there is also an opportunity for residents to serve as teachers themselves. However, busy clinical duties and the possibility of workflow interruptions among trainees are frequently mentioned deterrents to providing access to such opportunities [14]. Recognizing this, along with the difficulty for undergraduate students to shadow physicians due to increasingly stricter institutional rules, ESP was founded for undergraduates to shadow pediatric residents while assisting them in a non-physician level capacity. These clerical duties (i.e., organizing forms, faxing documents, scanning outside records, etc.) are not physician-level tasks, but within the outpatient clinical setting have been traditionally placed on residents and thus represent important elements in the clinical workflow. Assisting with these non-direct patient care related functions was incorporated into this shadowing program, and from the development of this symbiotic relationship, opportunities to mentor and teach without sacrificing resident workflow contributed to the acceptance and implementation of this program.

At the completion of their year-long ESP engagement, all student observers gained confidence in pediatric clinical care concepts. This shadowing experience was developed as a longitudinal commitment focused on increasing awareness of physicians’ clinical duties and addressing knowledge gaps regarding primary care. Significant exposure to pediatric patient care is often limited. Therefore, the scope of the shadowing experience included well child checks as well as urgent care visits in order to provide ample exposure to common pediatric care. Quarterly case conferences that allowed the learners to present patient cases in a formal setting to their peers are analogous to the patient presentations that the learners would later be making in medical school and beyond.

There are limitations to interpreting our questionnaire results. Most notably, the ESP undergraduates who participated were recruited from AMSA, and thus at study entry, each presumably already had a strong interest in medicine to be a member of a pre-medical organization. Nevertheless, there was still a statistically significant increase in students’ medical career interests from baseline. Over the 30-week program, the establishment of mentor-mentee relationship was less apparent. This was most directly identified in the resident questionnaire item scores, where most resident physicians felt neutral in regards to their ability to mentor the undergraduates during their time in clinic, as well as in responses made in the open-ended questions. Because of the ESP shift structure, each student typically interacted with an individual resident for only one four-hour shift. This limited one-on-one time may have produced a potential barrier to mentor-mentee relationship building as a result of limited time with each individual resident for lengthy career guidance discussions.

ESP participant growth was clearly evident in both the pediatric clinic and resident specific responses surrounding knowledge about the clinical duties of resident physicians, how a clinic functions, what an urgent care visit entails, and familiarity with well child checks. Thus, such a program like ESP, therefore, may have the potential to engage an early career interest in the field of pediatrics. Similar results have been documented from other specialty-specific shadowing programs including, anesthesiology and surgery [15-17].

Future steps for ESP will incorporate suggestions for improvement provided by the inaugural cohort of undergraduate students and pediatric resident trainees. Structured programmatic learning objectives will be developed and provided to both the students and residents, since more detailed roles for each party during observed patient encounters was noted to be an area of potential improvement. Learners will be expected to complete specific learning activities, such as how to document a patient encounter and present a case to an attending physician. Residents will be provided with the ESP program goals and a list of tasks that can be delegated to the undergraduate students, thereby ensuring transparency and consistency of expectations from both participant sides. Finally, future studies should directly evaluate the impact of the undergraduates’ involvement with non-direct patient care duties on the residents’ ability to better balance patient care and education while on rotation at this clinic, which has received negative impact from resident trainees in the past for having an imbalance between service and education.

## Conclusions

The introduction of this undergraduate-resident shadowing program addresses the knowledge gaps and clinical barriers revealed in pre-participation questionnaires. The experience thus far demonstrates the successful implementation of a shadowing program in a busy university outpatient clinical setting. This program illustrates an example of a symbiotic relationship, in which premedical students can gain greater insight into the clinical duties that physicians perform while providing the pediatric residents with the opportunity to teach and mentor. In addition, shadowers help balance service and learning for the residents by alleviating some of the non-physician level tasks routinely assigned to resident physicians. In summary, the implementation of this program demonstrates that such a formal shadowing program can benefit both the undergraduate learners and resident teachers with little to no obstruction of clinic workflow. Such a formal symbiotic shadowing program in a clinic can be easily replicated across other departments and institutions.
